# Lightning‐Induced Keraunoparalysis and Rhabdomyolysis: A Case Report

**DOI:** 10.1002/ccr3.71605

**Published:** 2025-12-05

**Authors:** Sushmita Bhattarai, Sagun Baral, Khem Chandra Joshi

**Affiliations:** ^1^ Department of Internal Medicine Rapti Academy of Health Sciences Ghorahi Nepal

**Keywords:** hypokalemia, keraunoparalysis, lightning, neurological complications, rhabdomyolysis

## Abstract

Lightning‐induced keraunoparalysis is a transient, reversible paralysis that can mimic spinal cord injury, while rhabdomyolysis—even without renal impairment—may coexist. Early recognition, vigilant monitoring, and correction of electrolyte disturbances such as hypokalemia are crucial. This case, reported from a resource‐limited setting, underscores the importance of timely conservative management to ensure full recovery and prevent unnecessary interventions or life‐threatening complications.

## 1 Introduction

Lightning occurs when electrical charges move either between clouds or between a cloud and the ground. This happens when the voltage difference exceeds 30,000 V, overcoming the natural resistance of the air [1]. Consequential lightning strikes (CLS) refer to lightning incidents that lead to physical injuries, loss of consciousness, or death in humans [1]. Lightning can cause injury through six primary mechanisms: direct strike, flash discharge (also known as splash), contact injury, side flash, ground current or step voltage, blunt force trauma, and upward streamers [2]. Injuries sustained due to lightning strikes result in a global mortality rate ranging from 0.2 to 0.8 per million people annually [2].

Almost any organ system can be affected by lightning strikes. Rhabdomyolysis, the breakdown of skeletal muscle and release of muscle contents, can range from mild, asymptomatic elevations of Creatine kinase (CK) to severe, life‐threatening complications, including electrolyte imbalances, acute kidney injury, and disseminated intravascular coagulation [3]. Lightning strikes can cause diverse neurological problems, including seizures, movement disorders, paralysis, sensory disturbances, and autonomic dysfunction [4]. Keraunoparalysis (KP) is a transient paralysis that can occur in certain individuals after a lightning strike, believed to be secondary to massive stimulation of the autonomic system [5]. We present the case of a 35‐year‐old male who was struck by lightning, resulting in thermal burns and temporary flaccid weakness of bilateral lower limbs and the left upper limb. He experienced a persistent elevation in creatine kinase levels, suggesting rhabdomyolysis, and was managed prophylactically. With conservative treatment, he achieved full neurological recovery without any renal complications.

## 2 Case History/Examination

A 35‐year‐old previously healthy male farmer was brought to the emergency department of Rapti Academy of Health Sciences in Dang, Nepal, after being struck by lightning while working in an open field during a thunderstorm. According to eyewitnesses, the patient collapsed immediately after the lightning strike and remained unresponsive for approximately 2–3 min before being transported to the hospital by coworkers.

On arrival, the patient had regained consciousness and was alert and oriented to time, place, and person. He complained of severe burning pain in the right thigh and reported acute bilateral lower limb and left upper limb weakness. Vital signs were within normal limits: blood pressure 130/80 mmHg, heart rate 102 bpm (regular), respiratory rate 18 breaths per minute, oxygen saturation 97% on room air, and axillary temperature 36.8°C.

Pulses in the bilateral lower limbs were diminished, and the limbs were swollen, cool, and pale.

Neurological examination demonstrated reduced motor strength: the lower limbs showed power of 3/5 on the left and 1/5 on the right, while the left upper limb had a strength of 3/5 and the right upper limb was normal, as assessed using the Medical Research Council (MRC) grading scale. Tone was decreased in the bilateral lower limbs and the left upper limb. Plantar reflexes were bilaterally mute, and deep tendon reflexes were diminished. Sensory modalities and cranial nerve function were intact. Remarkably, the left upper limb weakness improved within 6 h. Furthermore, power in the bilateral lower limbs gradually improved within 12 h. This transient and reversible paralysis was consistent with keraunoparalysis. Though no imaging or neurophysiological studies were performed, the rapid clinical recovery and lack of neurological sequelae supported the diagnosis.

**FIGURE 1:**
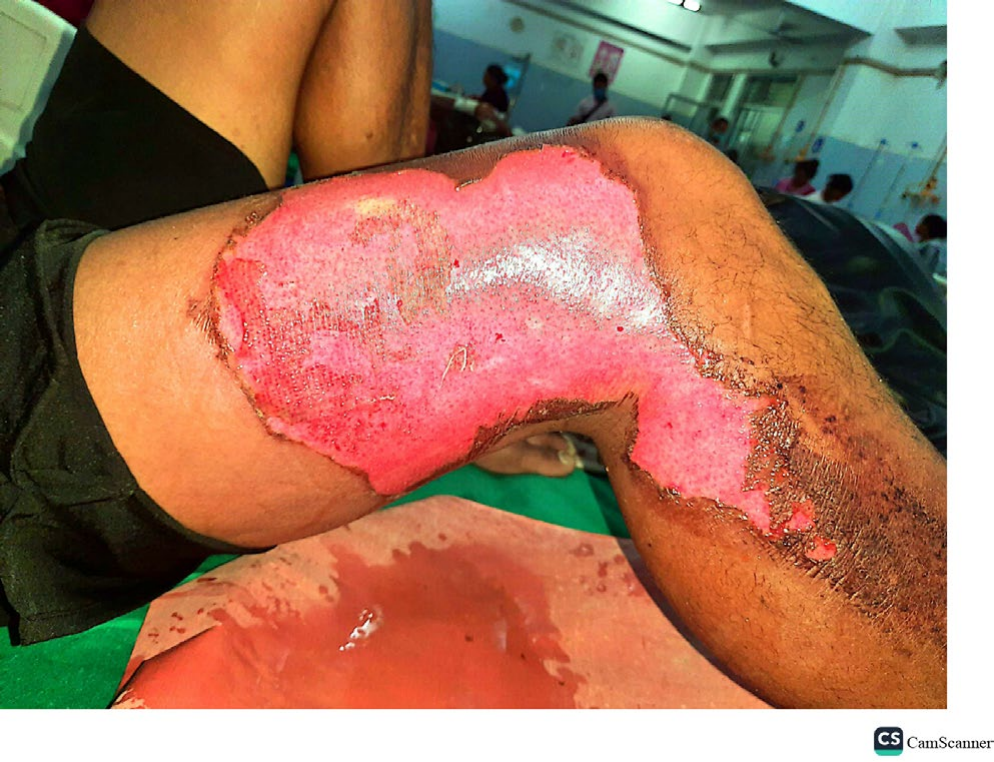
Lightning‐induced partial‐thickness burn on the right thigh.

Cutaneous examination (Figure 1) revealed a large, irregular partial‐thickness burn over the anterior aspect of the right thigh, extending to the proximal right leg. The lesion displayed erythema, epidermal sloughing, and a moist, pink underlying dermis consistent with second‐degree burns. At the lesion's periphery, well‐demarcated hyperpigmentation and dry eschar were observed. No charring, deep tissue necrosis, or pathognomonic Lichtenberg figures were present.

## 3 Investigations and Treatment

Laboratory evaluation (Table 1) revealed a significant elevation in serum creatine kinase, peaking at 10,612 U/L on Day 2. The patient remained hemodynamically stable, with preserved urine output and clear urine, and showed no clinical or biochemical evidence of AKI throughout the hospital stay. Renal function parameters remained within normal limits, and urine myoglobin testing on Day 2 was negative. Hypokalemia (2.8 mmol/L) noted on admission was corrected with intravenous supplementation. Other laboratory values, including serum electrolytes, complete blood count, cardiac enzymes, and urinalysis, were unremarkable.

**TABLE 1:** Trends in renal and muscle injury markers during hospital stay.

Day	Creatine kinase (U/L)	Potassium (mmol/L)	Urea (mg/dL)	Creatinine (mg/dL)	Urine myoglobin
1	4669	2.8	46.7	0.9	—
2	10,612	4.7	—	—	Negative
3	—	4.8	20.89	1.04	—
4	5447.71	—	—	—	—

The patient was admitted for close observation and supportive management. Initial treatment included fluid resuscitation, analgesia, tetanus toxoid administration, and local antiseptic wound care. Continuous cardiac monitoring revealed no arrhythmias. There was no evidence of internal injury, blast trauma, or fractures. Serial laboratory tests were obtained to monitor for potential complications, particularly rhabdomyolysis and acute kidney injury (AKI).

## 4 Outcome and Follow‐Up

Despite laboratory evidence of significant muscle injury consistent with rhabdomyolysis, the patient did not develop acute kidney injury or myoglobinuria, an uncommon but clinically significant variant in the spectrum of lightning‐associated rhabdomyolysis. The absence of renal impairment may be attributed to early hydration, mild myocyte damage, or rapid clearance of muscle breakdown products.

**FIGURE 2:**
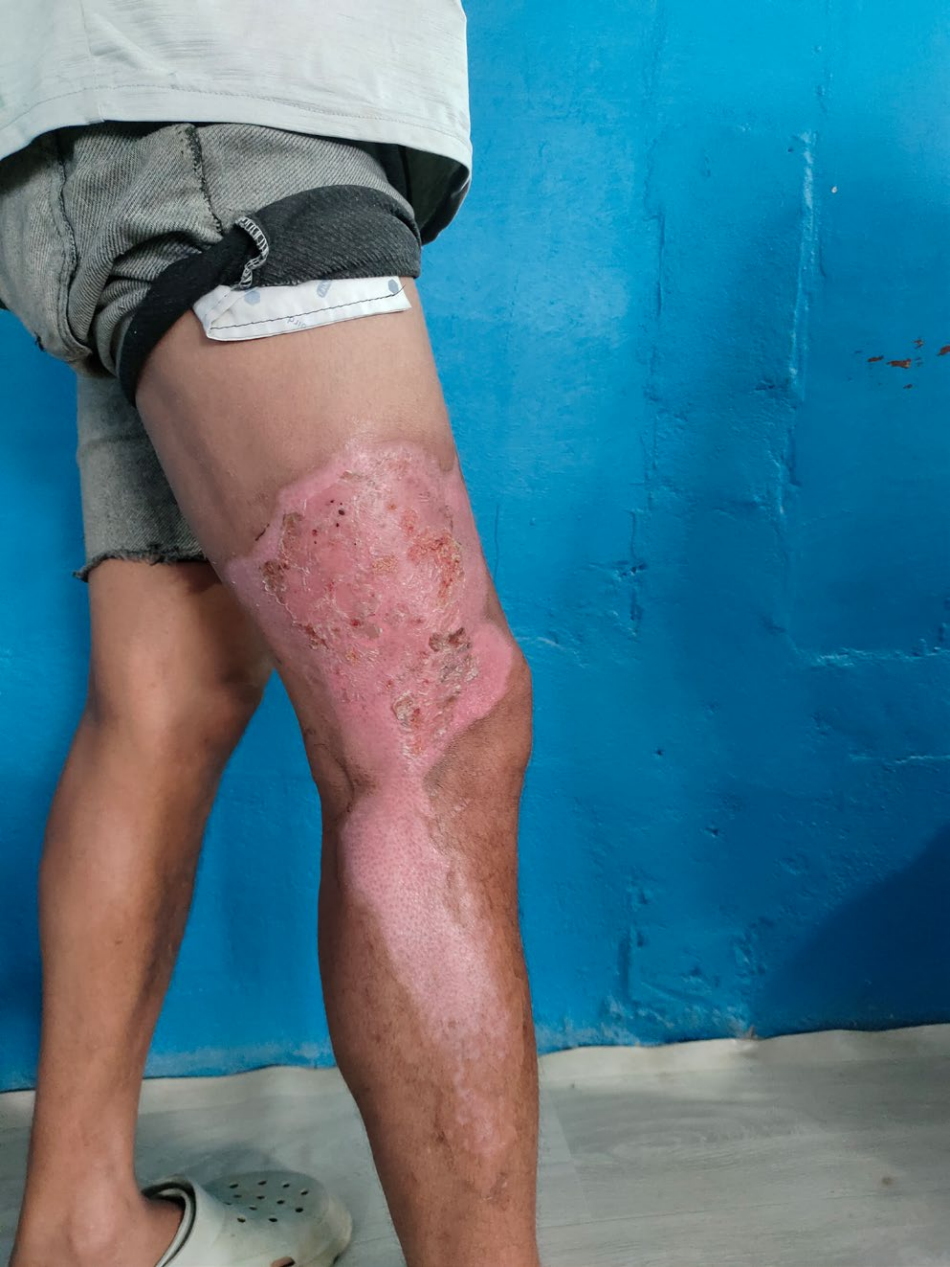
Healing burn wound and patient standing without support.

The patient was discharged on Day 6 with well‐healing burns, full neurological recovery, and normal renal function. At the two‐week outpatient follow‐up, the burn wound was healing (Figure 2); he remained asymptomatic with no residual deficits.

## 5 Discussion

Lightning is one of the deadliest natural disasters, estimated to cause thousands of deaths globally each year, with injuries occurring at a rate roughly 10 times higher [6]. Survivors of lightning strikes often experience a wide spectrum of clinical manifestations, ranging from superficial burns to complex neuromuscular, cardiovascular, and renal complications.

Our patient experienced a brief loss of consciousness and keraunoparalysis—two well‐documented, transient phenomena in lightning strike victims, conditions reported to occur in approximately 75% and 67% of lightning strike cases, respectively [6]. Keraunoparalysis is a temporary neurological condition that typically resolves within a few hours. It usually affects a limb through which the electrical current has passed, resulting in localized vasospasm, rendering the limb cold, pale, numb, and without a detectable pulse [4]. Its cause remains plausible and is believed to be secondary to massive stimulation of the autonomic nervous system rather than structural damage [4, 5]. Lower limb involvement is more common than upper limb involvement [6]. Keraunoparalysis may present in various forms, including hemiparesis [7], paraparesis [8], peripheral neuropathies, or others. In our case, the patient presented with acute bilateral lower limb weakness along with left upper limb weakness, diminished pulses, and cool, pale extremities following a lightning strike, consistent with a diagnosis of keraunoparalysis. The right upper limb remained unaffected, and the neurological deficits resolved spontaneously within 12 h, further supporting the transient and reversible nature of the syndrome. Although neuroimaging and neurophysiological studies were not performed, which may be a limitation, the clinical course was typical, and the absence of residual neurological deficits at discharge confirmed a benign outcome.

Another significant aspect of this case was the development of rhabdomyolysis, a condition characterized by the rapid breakdown of skeletal muscle tissue. Rhabdomyolysis in lightning injuries could result from the electrical impact of lightning, the intense heat it generates, and the concussive forces involved, all of which can cause muscle trauma [2]. Rhabdomyolysis can vary in severity, from a symptomless condition marked by elevated creatine kinase (CK) levels to a critical, life‐threatening state involving severely high CK, electrolyte disturbances, and acute kidney failure [3]. An increased creatine kinase (CK) level is the most sensitive laboratory indicator for detecting muscle injury that may lead to rhabdomyolysis [3]. Our patient exhibited a marked rise in serum CK, peaking at over 10,000 U/L, well above the diagnostic threshold. Despite this biochemical evidence of significant muscle injury, he did not develop acute kidney injury (AKI), likely due to prompt and adequate fluid resuscitation. Urine myoglobin was negative, and the patient's urine remained clear throughout hospitalization. While dark brown urine due to myoglobinuria is a classic early indicator of rhabdomyolysis, studies have shown that up to 26% of patients with rhabdomyolysis may not have detectable levels of urinary myoglobin, as was observed in this case [2].

Although the patient had hypokalemia (2.8 mmol/L) on admission, which can contribute to muscle weakness and paralysis, the rapid onset and transient nature of his limb weakness, along with the pattern of neurological deficits, were more consistent with keraunoparalysis. Therefore, while hypokalemia was considered in the differential diagnosis, it was unlikely to be the primary cause of the paralysis in this case. Correction of potassium was performed as a precautionary measure to prevent potential exacerbation of neuromuscular weakness.

The favorable renal outcome may also reflect the relatively mild degree of muscle necrosis or efficient renal clearance of myoglobin and other muscle breakdown products. Continuous monitoring and early intervention were essential in preventing progression to renal failure, a potentially fatal complication of rhabdomyolysis.

This case highlights the rare coexistence of lightning‐induced keraunoparalysis and rhabdomyolysis without renal impairment, emphasizing the importance of recognizing both neurological and systemic manifestations of lightning injuries. Timely supportive management—including correction of hypokalemia and close monitoring for rhabdomyolysis—facilitated complete neurological and renal recovery without invasive investigations. The case is particularly relevant in underreported, resource‐limited settings like Nepal, providing practical guidance for clinicians. While the absence of neuroimaging is a limitation, the rapid clinical recovery supports the diagnosis and benign prognosis. Early recognition of keraunoparalysis, careful differentiation from spinal cord injury, and vigilance for electrolyte disturbances and rhabdomyolysis can prevent unnecessary interventions and life‐threatening complications.

## Author Contributions

**Sushmita Bhattarai:** conceptualization, data curation, formal analysis, methodology, supervision, validation, visualization, writing – original draft, writing – review and editing. **Sagun Baral:** conceptualization, formal analysis, investigation, supervision, writing – review and editing. **Khem Chandra Joshi:** methodology, supervision, visualization, writing – review and editing.

## Funding

The authors have nothing to report.

## Ethics Statement

The authors have nothing to report.

## Consent

Written informed consent was obtained from the patient for publication of this case report and accompanying data.

## Conflicts of Interest

The authors declare no conflicts of interest.

## Data Availability

Data sharing not applicable to this article as no datasets were generated or analysed during the current study.
